# Which Way?

**Published:** 1885-07

**Authors:** 


### WHICH WAY?

The only complaint of the INDEPENDENT PRACTITIONER that has ever been made to us, was entered by a certain perambulating practitioner of circulatory peregrinations — in other words, a cat-whipper. His grievance was, that too many of the articles which it contained required study. They were above his comprehension. He wanted more amalgam and red-rubber. His longing was for specific instruction, in three words, of how infallibly to cure any oral disease that should come under his observation. We satisfied that yearning on the spot, and within the prescribed limits — "Pull it out;" that covered the ground. He was not likely to recognize more than one oral disease — toothache — and his comprehension would not include any other remedy than a mechanical one. Nor had he any very intense itching to improve his status, and to rise to a higher plane. His ideal conception of a professional journal was that of one which should give him everything in a nutshell. He only wanted the cream. He was pre-eminently one of those "busy practitioners," who are so driven by professional duties that they have no time for reading thoughtful articles. It was specifics that he was after; aphorisms — axioms — excerpts — *chestnuts*. If the I. P. would only condense and give pithy extracts, and confine itself to recipes and formulas and practical instructions, it would suit him better. What did he care for the etiology of disease? All he wanted was to know what would cure it. Of what interest to him was the pathological character of any condition? He had no time for these fooleries. He was too busy for such nonsense. But he would not mind the two dollars and a half, though money was very scarce with him, if we would only tell him how always to extract a tooth without breaking it off, or if we would give him some infallible remedy for "toothache," or publish a recipe that would invariably cure corns.

It is needless to say that we do not aim to publish that kind of a journal. The INDEPENDENT PRACTITIONER desires to be instructive; not amusing, or entertaining. If the dentist desires a jest-book, let him subscribe for "Puck," or "Life," or "The Judge." If he seeks to be entertained, he should read "The Atlantic," or "The Century," or "The New York Ledger;" his choice depending upon the quality of his literary taste. It is the aim of this Journal to raise the professional standard, and not to lower it. We desire that its perusal shall result in better dentists; that its subscribers shall be well informed upon professional matters and thoroughly intelligent concerning new methods of practice. We are laboring to educate, and elevate, and edify. Perhaps the success attained is problematical and the methods questionable, but no man can impugn the motives.

No one is enlightened or instructed by a continued study of things with which he is already familiar. Stock-breeders do not teach young horses to carry their heads high by allowing them to eat off the ground, but by placing their food at the extreme height of their reach. If teachers never got beyond the alphabet, their pupils would learn very little of literature. They lead the young mind on by continually setting new tasks that lie just beyond the limits of present knowledge. If this Journal were to present papers and publish discussions only upon such questions as the average dentist thoroughly comprehended, on matters that were easily within his reach, how much would it add to his store of information? Would he gain in professional intelligence, or improve in actual practice by the study of trite matters? The general tone of dentistry is only to be raised by elevating the status of the individual practitioners. To get them on higher ground can only be accomplished by leading them up hill. We would have every dentist standing on tiptoe, straining his gaze to catch the first dawn of a new Truth as it rises above the hill-tops of ignorance and prejudice.

So the Independent Practitioner is continually publishing articles and essays that are a little beyond the information of the average dentist; articles that require study, and investigation, and thoughtful consideration for their full comprehension; articles prepared by those who are specially qualified in their chosen department; articles conveying, perhaps, different views of the same subject; articles possibly contradictory in tone and statement, but all tending toward a more thorough understanding of professional matters, all assisting in the general diffusion of light by throwing each its ray of illumination upon a hitherto dark place.

It might be asked, of what profit is it to the average dentist to study the mycology of Dr. Miller, and to attempt to fathom all the meaning of his pure cultures of the bacteria of the mouth, or his investigations of their pathogenic character, or his studies of their morphology and proliferation ?

It is not expected that every dentist will make a special study of bacteriology, but every member of a liberal profession should possess a reasonable amount of intelligence concerning the scientific aspect of his calling. The researches of Dr. Miller have brought us as a profession to higher ground, but the road has of necessity led up a steep declivity, and the path has been a rugged one. When he first essayed it there were comparatively few men in America who comprehended where it was to lead, but the number is daily growing of those who take a broader view of the matter, and who listen respectfully when men who are not mere speculative theorists, but who pursue original investigations, talk to us of matters heretofore only dimly apprehended.

In our May number Dr. Ross gave a thoughtful article upon Epithelioma. What proportion of the dentists of the country possessed sufficient information upon this subject, either to give an enlightened assent to his statements or to understandingly differ ? By the average "busy dentist," who has "no time for reading," the article was only cursorily examined. But even to see the title in the table of contents starts a train of reflection, more or less permanent in character, through his mind. He knows that it is a subject upon which he should be informed, and his attention is attracted to it. Perhaps he goes back and does read it through, with only a partial insight into the lesson that it was desired should be conveyed, but it is all in the line of progress. His eyes are becoming accustomed to the light, and he commences to realize that the time must come when he, as well as Dr. Ross, should be competent to treat all such lesions as the ones described. Perhaps such an article may not attract the attention that is its due ; some poor, untaught, illiterate, narrow-minded soul may even cry out against its appearance, as foreign to *his* dentistry, and as too scientific and erudite. What then ? It has served its purpose, and a good purpose too, in giving an electric shock to these half-dentists. It may not teach them much, but it will at least wake them up. Another opening for the admission of light has been made, and soon the illumination will be perfect.

Too many of our number treat dentistry as if it were wholly a mechanical pursuit. At society meetings they may prate loudly of the dignity of dentistry, and claim membership in a *profession*. But their practice and their general conduct is that of mechanics. For instance — they are continually seeking after specifics. They desire to work by rule and square; to proceed according to exact mechanical directions. Like our friend of the first paragraph, they want axioms, and precepts, and recipes, and infallible laws. They do not comprehend that these things belong to a mechanical trade. Only half educated, and without the patience and studious habits that would enable them to thoroughly investigate any subject, they wish to arrive at the desired end by unerring rules and patent methods. In medicine they want a comprehensive remedy, that shall cure every known ill. They do not reflect that a *profession* deals with principles and general laws, which require a professional education and special training that they may be intelligently applied. They will not spend the time to carefully study an article that properly presents these things, but would jump at the conclusion by glancing at an unintelligible abstract, and so they go through life, trying to adapt mechanical laws to professional questions, and meet with a well-deserved failure.

It is needless to say that the Independent Practitioner will not cater to such tastes, but will, to the extent of its ability, labor to establish a better order of things, and to lead men to a higher plane, even though it be infinitely harder work to climb up the hill than to pursue the beaten level of the common-place. As for reprinting articles from other journals, we do very little of that. Not that we would be unable to find excellent matter to copy in our contemporaries, but because that so long as this Journal has a distinct voice of its own we prefer to use it, even though it be not so thrilling, rather than to copy the tones of the biggest lion that roars. We usually have enough of original material to serve our purpose, and what is presented in these pages will not be found elsewhere.

